# Steigerung der Innovationskraft einer Schweizer Stiftung – IdéeSport’s Transformation zu einer agilen Organisation

**DOI:** 10.1365/s40702-023-00978-w

**Published:** 2023-05-10

**Authors:** Katja Kurz, Ninja Leikert-Boehm, Christian Russ, Marcel Roland Sieber

**Affiliations:** 1 ZHAW School of Management and Law, Theaterstraße 17, 8400 Winterthur, Schweiz; 2 Department of Management, Prague University of Economics and Business, W. Churchill Sq. 1938/4, 130 67 Prague 3, Tschechien

**Keywords:** Agile Transformation, Innovation, Stiftung, Non-Profit-Organisation, Agile transformation, Innovation, Foundation, Non-profit organization

## Abstract

Die agile Transformation kann ein Weg für Unternehmen sein, ihre Innovationskraft zu stärken und dabei ein Gleichgewicht zwischen radikal erneuernden und laufend optimierenden Lösungen für aktuelle Herausforderungen zu finden. So sah auch die Schweizer Non-Profit-Organisation IdéeSport ihre Chance, sich auf agile und selbstorganisierte Weise zu transformieren, um für ihre Zielgruppen attraktiver zu werden. Basierend auf dem HR Pioneers Trafo Model und dem Kreismodell kollegialer Führung wendete IdéeSport eine praxisnahe Transformationsarchitektur mit Handlungsclustern in drei aufeinanderfolgenden Phasen an. Dieser Prozess wurde durch einen Fallstudienansatz mittels Durchführung von Workshops und Interviews wissenschaftlich begleitet. Die Aktionsforschung des Unternehmens zeigte, wie wichtig eine schrittweise Planung und Umsetzung ist, die Kunden, Mitarbeiter und Führungskräfte von Anfang an einbezieht, und wie entscheidend es ist, eine organisch gewachsene Lernkultur zu etablieren. Die vorliegende Arbeit zeigt auf, welche Handlungscluster für die erfolgreiche Transformation von IdéeSport in eine agile Organisation relevant waren, und formuliert praktische Handlungsempfehlungen für eine agile Transformation im öffentlichen Stiftungswesen in der Schweiz.

## Abstract (en)

Agile transformation can be a way for companies to strengthen their innovative power while finding a balance between explorative and exploitative solutions to current challenges. This is how the Swiss non-profit organization IdéeSport saw its opportunity to transform in an agile and self-organized way to become more attractive to its target groups. Based on the HR Pioneers Trafo Model and the circle model of collegial leadership, IdéeSport applied a practical transformation architecture with action clusters in three successive phases. This process was scientifically accompanied through a case study approach by conducting workshops and interviews. The company’s action research showed the importance of step-by-step planning and implementation that involves customers, employees, and managers from the beginning, and how crucial it is to establish an organically grown learning culture. This paper identifies which action clusters were relevant to IdéeSport’s successful transformation into an agile organization and formulates practical recommendations for an agile transformation in the public foundation sector in Switzerland.

## Ausgangslage

In der Schweiz durchlaufen bereits eine Vielzahl von Unternehmen, speziell in der IT, eine Transformation hin zu höherer Agilität. Laut der Studie von Futurum (2019) sehen 82 % der befragten Chief Executive Officers (CEO) Schweizer kleiner und mittlerer Unternehmen (KMUs) einen ganzheitlichen agilen Ansatz als Erfolgsfaktor. Jedoch gibt es noch immer Herausforderungen auf dem Weg dorthin, wie zum Beispiel unflexible Entscheidungsmechanismen auf verschiedenen Organisationsebenen, Rollenunklarheiten oder anfängliche Überforderung (Majkovic et al. 2019, S. 13). In der Studie zu agilen Arbeitswelten und Organisationsformen in der Schweiz von Majkovic et al. (2019) wird klar dargestellt, dass diese auf der Ebene Mitarbeitende, mittleres Management und oberes Management bestehen.

Nicht nur wirtschaftliche Unternehmen sehen Chancen in agilen Organisationsformen (Tscherne et al. 2018). Die Schweiz verfügte 2021 über 4860 offiziell kontrollierte Stiftungen (Eidgenössisches Departement des Innern EDI). Eine spezifische Form von Stiftungen stellt die Non-Profit-Organisation (NPO) dar. Diese zeichnet sich durch ihre Philanthropie, Gemeinnützigkeit und altruistisches Engagement zugunsten der Allgemeinheit aus (SwissFoundations 2021). Trotz deren Ausrichtung und hohen Motivation, welche zumeist in solchen Organisationen vorherrschen, sind agile Führungskonzepte noch wenig verbreitet (Simsa et al. 2013; Klimčíková 2018).

Im konkreten Fall wird die Schweizer NPO IdéeSport vorgestellt, welche den neuartigen Schritt in Richtung agiler Selbstorganisation gewagt hat. Das Ziel war es, den Mitarbeitenden neue Handlungsspielräume zu geben, der zuletzt gestiegenen Fluktuation entgegenzuwirken und damit die Innovationskraft, als auch die Service- und Kundenorientierung zu erhöhen. Dafür wurde ein systemischer Ansatz der Organisationstransformation von einer klassischen zur agilen Organisation vorangetrieben. Vision, Prozesse, Rollen und Organisationsstrukturen wurden mittels agiler Methoden neu definiert, um von der für NPOs typischen intrinsischen Motivation und Einsatzbereitschaft profitieren zu können. Zusätzlich wurden die neuen Ansätze inkrementell umgesetzt und wissenschaftlich beobachtet. Da dieser ganze Veränderungsprozess im laufenden Betrieb gemeistert wurde, benötigte es eine gute Balance zwischen dem Fortbetrieb und Ausbau der Dienstleistungen, als auch dem explorativen Entwickeln von neuen Lösungsformen. Somit war der Weg selbst agil, als auch das Ergebnis eine agile Organisation, welche flexibler und resistenter auf zukünftige Anforderungen einer NPO reagieren kann. Schlussendlich resultierte daraus eine Prozessinnovation mit der Herausforderung der Ambidextrie, welche mit einer Produkt- und Serviceinnovation kombiniert, gemeistert wurde. Die massgeblichen Schritte der Transformation und ihrer Kernelemente, wie auch die Erkenntnisse, wurden in Leikert-Boehm et al. (2022) detailliert dokumentiert. Diese Fallstudie hat das Ziel, die wesentlichen Handlungsempfehlungen, auch aus Sicht der Innovationskraft, aufzuzeigen.

Im Auftrag von IdéeSport wurde ein Projekt zur wissenschaftlichen Begleitung der Bestrebungen der agilen Transformation durchgeführt. Die Ergebnisse werden in diesem Artikel anhand der folgenden Forschungsfrage vorgestellt: *Welche wesentlichen Handlungscluster waren für die erfolgreiche Transformation von IdéeSport in eine agile Organisation relevant?*

## Die Fallstudie IdéeSport

Die Schweizer Stiftung IdéeSport hat sich seit 1999 der Suchtprävention, der Gesundheitsförderung und der gesellschaftlichen Integration im Bereich der Kinder- und Jugendförderung durch Sport als Ziel gesetzt. Die Auftraggeber der Stiftung setzen sich aus den schweizerischen Kantonen und Gemeinden zusammen. Die Finanzierung der IdéeSport erfolgt durch Beiträge vom Bund, den Kantonen und den Gemeinden sowie zusätzliche Spenden und Unterstützung durch andere Stiftungen.

Die IdéeSport bietet verschiedene Programme für Kinder, Jugendliche und junge Erwachsene an, die für die Teilnehmenden kostenlos und unverbindlich sind. Das Angebot erstreckt sich mit bis zu 165 Standorten über die ganze Schweiz und umfasst keine leistungsorientierte Sportgestaltung. Es geht um ein polysportives Miteinander als gemeinsames Erlebnis, teils auch unterstützt mit Musik oder gestaltet als freies Spiel in Erlebnislandschaften (IdéeSport).

Um den stetigen Veränderungen des Marktes gerecht zu werden, entschied sich IdéeSport im Mai 2019, die eher klassische, statische Organisation in eine agile Organisation zu transformieren (IdéeSport 2020; Antonello 2020). Damit in den Projekten schneller und direkter auf die Bedürfnisse ihrer Zielgruppen reagiert werden kann, entschied sich IdéeSport für die agile Transformation der Organisation und für Ansätze der kollegialen Führung.

In der Schweiz war IdéeSport mit diesem Schritt eine der ersten NPOs, die den Weg zur Selbstorganisation gewagt hatte.

## Theorie und Methode

### Theoretische und praktische Grundlagen

In der vorgestellten Fallstudie wurde für die agile Transformation selbst auch Scrum verwendet, um in Richtung einer ersten agilen Organisationsform zu gelangen. Der Ursprung von „agilen Methoden“ ist bereits in den 1970ern in der Softwareentwicklung zu erkennen. Im Jahre 2001 wurde das „Agile Manifest“ geboren, mit dem Ziel kundenorientiertere Software zu kreieren (Beck et al. 2001). Dabei wird agiles Denken propagiert, welches auf Zusammenarbeit, Selbstorganisation und der Akzeptanz von Veränderung basiert. Als eine Weiterentwicklung der agilen Methoden kristallisieren sich die noch nicht scharf definierten agilen Organisationen heraus (Renzl et al. 2021). Für diese Arbeit werden sie als neue Form der Organisationsstruktur, Führung und Kultur gesehen, welche besser mit Unsicherheit, Veränderung und anderen Dynamiken in ihrem Anwendungsbereich umgehen kann (Anderson und Uhlig 2015; Gloger und Margetich 2018).

Ein wesentliches agiles und leichtgewichtiges Softwareentwicklungs-Vorgehensmodell ist Scrum (Schwaber und Sutherland 2020). Die Scrum-Methode der iterativen Sprints, als vorgegebene Zeiteinheit zur Erarbeitung gewisser Ziele, wird heute zunehmend auch außerhalb der Softwareentwicklung eingesetzt (Gloger und Margetich 2018; Preußig 2018).

Speziell im Bereich der verbesserten Produktivität und Innovationskraft wird agilen Vorgehen ein hoher Mehrwert zugeschrieben (Oestereich und Schröder 2017; Krstić et al. 2018; Findsrud 2020). Nachhaltiger Erfolg einer Transformation bedeutet jedoch nicht nur, völlig flexibel zu sein und sich ständig zu verändern. Gemäss Studien von Raisch et al. (2009) und O’Reilly und Tushman (2013) ist ein Gleichgewicht zwischen Flexibilität und Beständigkeit in einer erfolgreichen Organisation erforderlich. Diese von O’Reilly und Tushman (2013) als organisatorische Ambidextrie beschriebene Fähigkeit ermöglicht einer Organisation, ein Gleichgewicht zwischen der Erkundung neuer Ansätze und der damit verbundenen Annahme von Unsicherheit und der kontinuierlichen Nutzung vorhandener Stärken und Notwendigkeiten zu finden (He und Wong 2004).

**Abb. 1:**
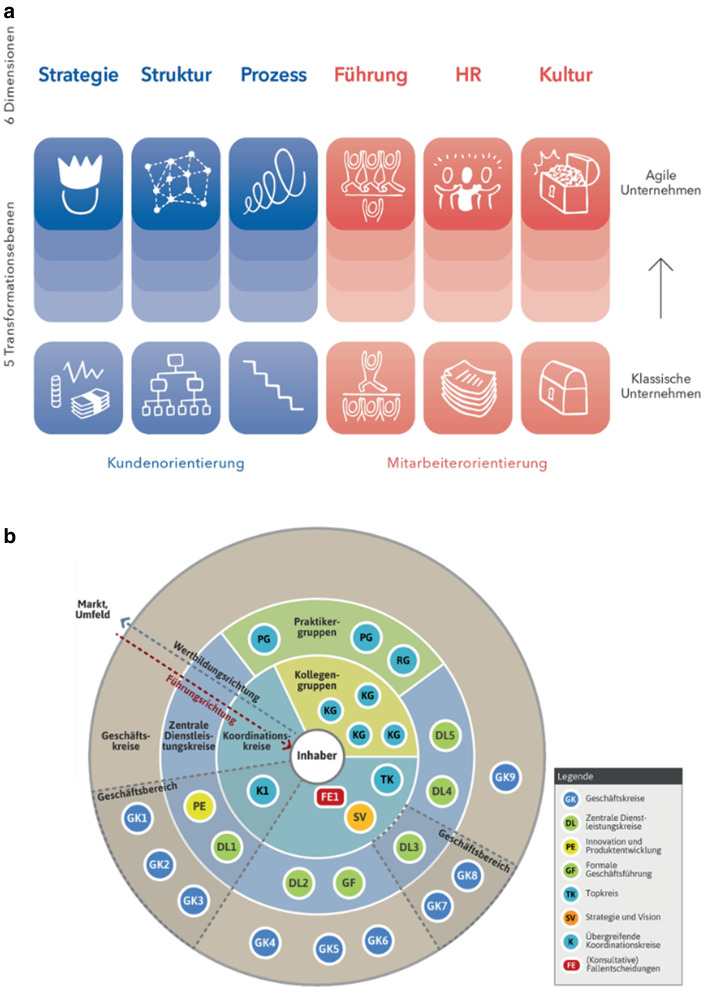
Eingesetzte Modelle – Pioneers Trafo-Modell^TM^ (Lehmann et al. 2021, S. 9) und kollegiales Kreismodell nach Oestereich und Schröder (2017, S. 80)

Um eine agile Transformation zu initiieren und voranzutreiben, bedarf es umsetzbarer und verständlicher Vorgehensmodelle. Ein Modell, welches von IdéeSport identifiziert wurde, ist das von HR Pioneers entwickelte Trafo-Modell (Häusling, 2020). Laut Häusling (2020) sollte das Unternehmen bestrebt sein, die Veränderung zur agilen Organisation über die sechs Dimensionen des Trafo-Modells in Einklang zu bringen (vgl. Abb. 1a).

Als weiteres Modell zur Förderung der Agilität und damit der Innovationskraft entschied sich IdéeSport das sogenanntes Kreismodell nach Oestereich und Schröder (2017, S. 80) anzuwenden. Dabei wird das Prinzip der Kollegialen Führung nach Oestereich und Schröder (2017) in Form eines kreisförmigen Organisationsmodells für agile Strukturen eingesetzt (vgl. Abb. 1b). Die Führung wird zum einen als Instrument betrachtet, zum anderen als die Struktur der Organisation (Oestereich und Schröder 2017, S. 25 ff.). Der Vorteil des Modells besteht darin, dass anhand dieser Organisationsstruktur dynamisch und innovativ auf Veränderungen im Markt und auch innerhalb der Gesamtorganisation reagiert werden kann (Oestereich und Schröder 2017, S. 81 ff.).

### Vorgehen der wissenschaftlichen Begleitforschung

Das Forschungsdesign folgt einem explorativen Ansatz, um mittels einer Fallstudie komplexe Zusammenhänge und sich abzeichnende Prozessergebnisse aufzudecken (Langley 1999; Eisenhardt und Graebner 2007). Dieser Ansatz wurde gewählt, um bessere Einblicke in die Transformation einer Non-Profit-Organisation in Richtung einer agilen Organisation zu erlangen. Dabei gliederte sich das wissenschaftliche Vorgehen in vier methodische Schritte, die detailliert in Leikert-Boehm et al. (2022) beschrieben sind:

1. Teilnahme und Beobachtung an Workshops und Sprint-Sitzungen des Agilitätsprojektteams, qualitative Auswertung (67,5 h)
2. Gruppeninterview mit dem Agilitätsprojektteam, qualitative Auswertung (10 h).
3. Interviews mit den internen und externen Experten, qualitative Auswertung (8 h).
4. Interne Umfrage für Einblicke in den agilen Reifegrad, quantitative Auswertung (3,5 h).

Das Monitoring des Transformationsprojekts dauerte 16 Monate von Mai 2020 bis September 2021. Drei Workshops konnten vor Ort durchgeführt werden, alle anderen Besprechungen, Interviews und Meetings fanden aufgrund der COVID-19-Pandemie online statt. Weitere gut 30 h flossen in die Dokumentenanalyse sowie in die Präsentation von Ergebnissen, d. h. es wurden insgesamt 120 Forschungsstunden aufgewendet.

## Transformationsarchitektur und Handlungscluster bei IdéeSport

**Abb. 2:**
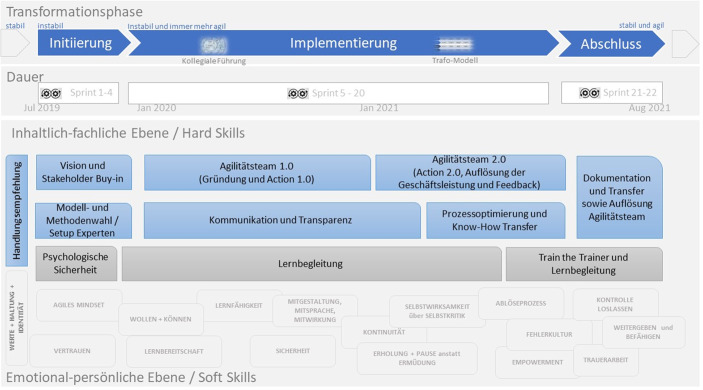
Transformationsarchitektur

Zur Orientierung und Übersicht des agilen Gesamtvorhabens dient die Transformationsarchitektur in Abb. 2. Diese bettet die relevanten Handlungscluster im Kontext vom zeitlichen Verlauf der Transformation, der inhaltlich-fachlichen Ebene sowie der emotional-persönlichen Ebene ein.

### Transformationsphasen und Dauer

Die Transformation war in drei Phasen gegliedert. Gerade im dynamischen Umfeld achtete IdéeSport präzise darauf, dass sich nicht alles permanent in Bewegung befand. Dieses bewusste Managen von Ambidextrie setzte IdéeSport während des kompletten Prozesses ein. Von einer stabilen Ursprungsorganisation kommend, begann die Stiftung mit einer instabilen Phase der Initiierung. In dieser Phase der hohen Veränderungsdichte etablierten sie die agilen Arbeitsweisen und Organisationsstrukturen durch inkrementelle Implementierungsschritte. Die Abschlussphase war gekennzeichnet durch Stabilisierung anhand kontinuierlicher, agiler Arbeit innerhalb der neu geschaffenen Rahmenbedingungen.

Der nahezu zwei Jahre andauernde Transformationsprozess der IdéeSport startete im Juli 2019 mit einem mehrtägigen Wissenstransfer-Workshop. Erst im Anschluss erfolgte die gemeinsame, bewusste Entscheidung für die Transformation. Sprint 1–4 prägten die Initiierung und dienten der Vorbereitung. Über den Verlauf der Transformationsphase gab es Phasen, in denen viel in sehr kurzer Zeit umgesetzt wurde und Phasen der Unsicherheit und Ermüdung. Wo nötig, nahm IdéeSport bewusst Tempo heraus und setzte sogar Sprints aus. Dies diente dazu die Agilität im Betriebsalltag zu integrieren und Fokus zu erhalten, ganz nach dem Prinzip der agilen Nachjustierung. Mit dem Sprint 21 im Frühsommer 2021 begann der Abschluss und endete mit dem Ritual einer Abschlussfeier und symbolischer „Übergabe“ vom Agilitätsteam an den Kulturkreis im August 2021.

Die Handlungscluster unterscheiden sich über die Wirkung der bewussten inhaltlich-fachlichen Ebene (blau) und der tieferliegenden, teils unbewussten emotional-persönlichen Ebene (grau). Die Wirkungsebene zeigt an, ob es sich in Anlehnung an Schein und Schein (2017) mehrheitlich um den unbewussten oder bewussten Bereich handelt.

Die Synthese in Form von den 10 Handlungsclustern fasst die Notizen, Protokolle, Abschriften und Präsentationen auf der Metaebene prägnant zusammen. Die verdichteten Handlungscluster sind in Tabellen mit den Abschnitten „Ziele“, „Wirkungsebene“, „Umsetzung“ und „Erkenntnisse“ dargestellt (Tab. 1–10).

### Initiierungsphase

Die Handlungscluster der Initiierungsphase mit den zugehörigen Werten des Vertrauens und der agilen Mindset-Arbeit legten den Grundstein für die Implementierung.

**Tab. 1:** Cluster Vision und Stakeholder – Buy – In

Vision und Stakeholder-Buy-In	
Ziel(e)	Orientierung/gemeinsames Verständnis schaffen Führungskoalition bilden und Sensibilisierung für die kommende Transformation
Wirkungsebene	Bewusst
Umsetzung	HR als Treiber der Transformation/Organisationsentwicklung sensibilisiert zunächst Stiftungsrat und Geschäftsleitung für die Transformation durch Aufzeigen der Dringlichkeit und Notwendigkeit. Das Management versteht, was das Ziel ist: – Klares Engagement, Verantwortung/Führung abzugeben – Vertrauen ist geschaffen/Lernkultur im Fokus Mitarbeitende einbeziehen: Das Transformationsprojekt wird als ein Projekt der Organisation und nicht der Geschäftsleitung lanciert; Entscheid zur Agilität fällt an einem Workshop von allen Mitarbeitenden
Erkenntnisse	Umfassende Diagnose und Analyse der zu optimierenden Situation nötig Der Paradigmenwechsel in der Führung muss akzeptiert und verstanden werden

**Tab. 2:** Cluster Modell- und Methodenwahl/Setup Experten

Modell- und Methodenwahl/Setup Experten	
Ziel(e)	Struktur/Orientierung aus (in der Praxis erprobten) Modellen Starten mit gefülltem Methodenkoffer
Wirkungsebene	Bewusst
Umsetzung	Metamodell Pioneers Trafo-Modell^TM^ (Häusling 2020) Kollegiale Führung/Kreisorganisation (Oestereich und Schröder 2017) Coaching und kontinuierliche Lernbegleitung durch Experten
Erkenntnisse	Die Selbstorganisation und Eigenverantwortung der Teams stehen im Mittelpunkt

**Tab. 3:** Cluster Psychologische Sicherheit

Psychologische Sicherheit	
Ziel(e)	Psychologische Sicherheit durch klar definierte Rahmenbedingungen/Transparenz Vertrauensaufbau/Unsicherheiten minimieren Widerständen/Ängsten frühzeitig entgegenwirken
Wirkungsebene	Unbewusst
Umsetzung	Mindset/Bereitschaft zur Transformation (wie expliziten Kündigungsschutz und ausgesetzte Gehaltsverhandlungen) positiv beeinflussen, Start eines Piloten: ein selbstorganisiertes Team Aufbau von Transparenz durch klare Roadmap/Milestones Einbezug aller Mitarbeitenden durch gemeinsame Aktivitäten (teils in Arbeitszeit): z. B. Sport, Homeoffice-Hopping
Erkenntnisse	Stetiges Einüben von Wissen und Können (Lernkultur) Aktive Bearbeitung von Spannungen/Widerständen Frühe Erfolgserlebnisse aus dem Piloten bereits im Sept. 2019

### Transformationsphase

Die beschriebenen Vorbereitungen ermöglichten einen guten Start in die blauen, inhaltlich-fachlichen Handlungscluster der Transformationsphase. Bei der Haltung (graue Cluster der emotional-persönlichen Ebene) bestand der Fokus auf Lernbereitschaft und Lernfähigkeit, welche durch aktive Mitarbeit, Mitsprache und Mitwirkung etabliert wurden. Kontinuität half, die Wichtigkeit des richtigen Tempos wurde deutlich. Es war Ausdauer gefragt und das richtige Timing für die Erholungsphasen war entscheidend für die kritischen Momente der Ermüdung, der starken Ungeduld bzw. Selbstkritik.

**Tab. 4:** Cluster Agilitätsteam 1.0

Agilitätsteam 1.0: Gründung des Teams und agile Projektdurchführung	
Ziel(e)	Voraussetzungen, Transparenz schaffen, Agilität lernen Vertretung der Belegschaft sicherstellen Entfesselte Motivation kanalisieren. Agile Arbeitsmethoden erlebbar machen
Wirkungsebene	Bewusst
Umsetzung	– Agilitätsteam gründen: Mitarbeitende wählen das motivierte, interdisziplinäre Agilitätsteam Kritisch eingestellte Personen werden ins Transformationsteam eingeladen Agilitätsteammitglieder sind mit 10–20 % ihrer Arbeitszeit alloziert – Projektdurchführung mit agilen Methoden (Scrum/Kanban-Boards): Lernbereitschaft (Wollen) in Lernfähigkeit (Können) verwandeln Umgestaltung der Büroräumlichkeiten für agiles Arbeiten Dynamische Anpassung der Delegationsmatrix, Rollenkonstitutionen und des Kreismodells
Erkenntnisse	Neues Thema fördert, dass alle wissbegierig sind Neues einführen und in kurzen Iterationen umsetzen Weg der Transformation für jeden machbar gestalten Rollenbesetzung stärkenbasiert mit Bewerbungs- und Wahlverfahren

**Tab. 5:** Cluster Kommunikation und Transparenz

Kommunikation und Transparenz	
Ziel(e)	Sichtbare/spürbare Aktionen zum Informationsaustausch Unsicherheiten/Widerstände aufnehmen durch Mitgestaltung, -sprache, -wirkung
Wirkungsebene	Bewusst/unbewusst
Umsetzung	Roadmap gibt Klarheit über das Vorgehen Intranet-Beiträge, Information zum Stand der Arbeiten im Agilitätsteam (Lern‑)Videos, Feedback der Gesamtorganisation einholen Finanztransparenz durch eine inkrementell eingeführte, zentrale IT-Lösung
Erkenntnisse	Erfolge erkennen, innerhalb und ausserhalb der Organisation kommunizieren Mitarbeitende zur richtigen Zeit informieren

**Tab. 6:** Cluster Lernbegleitung

Lernbegleitung	
Ziel(e)	Unsicherheiten in Kompetenz wandeln Ausbau von Lernbereitschaft und Lernfähigkeit Von der Selbstkritik zur Selbstwirksamkeit
Wirkungsebene	Bewusst/unbewusst
Umsetzung	Wählbare Lernbegleitung: Team entscheidet, wann/welche Fehlerkultur bzw. Lernkultur festigen Verantwortung übernehmen Mut haben, Dinge auszuprobieren und reflektieren lernen Gemeinsame Prinzipien und Werte auf der Basis von Mut und Vertrauen
Erkenntnisse	Persönliche Entwicklung ermöglichen Wissen, dass das Agilitätsteam einen Vorsprung hat; andere Kreise nicht abhängen Lernen nie abgeschlossen

**Tab. 7:** Cluster Agilitätsteam 2.0

Agilitätsteam 2.0	
Ziel(e)	Sicherstellen von Ambidextrie. Erneuerung und Reife ermöglichen Beachtung der Energielevels des Agilitätsteams und der Gesamtorganisation
Wirkungsebene	Bewusst/unbewusst
Umsetzung	– Action 2.0: Dynamische Anpassung von Team/Formaten. Reife hin zu Stabilität, z. B. Bedürfnisse erkennen, Nein-Sagen können – Auflösung Geschäftsleitung Formal Übergang zur agilen Organisation fixieren. Mitglieder in sinnstiftende Rollen oder dem Geschäftsleitungskreis überführen – Feedback zur agilen Reife aus der Organisation Beachtung der Rollendauer und -entwicklung in den Kreisen zur Kontinuität und Empowerment. Offene Fehlerkultur, „Vorleben“ des agilen Mindsets
Erkenntnisse	Überzeugung auf dem richtigen Weg zu sein, aber Reife und Mut haben, Tempo zu drosseln, Übermüdung vermeiden Reife im Selbstmanagement Fluktuationsrate konnte halbiert werden

**Tab. 8:** Cluster Prozessoptimierung, Know-how-Transfer

Prozessoptimierung, Knowhow-Transfer	
Ziel(e)	Hinterfragen von Prozessen/Optimierung auf Organisationsstrukturen Selbstwirksamkeit anstatt Selbstkritik, Empowerment Aller
Wirkungsebene	Bewusst
Umsetzung	– Kern- und Supportprozesse Analyse und Optimierung iterativ, Fehler machen, anhand Erfahrungen nachbessern Verantwortlichkeiten gemäss angepassten Prozessen und Strukturen neu definieren – Pausierte Themen (Lohnmodelle, Kündigung) wieder aufgegriffen – Know-how-Transfer durch kontinuierliches Coaching und Weiterentwicklung
Erkenntnisse	Zeitgerechte Qualifizierung Beteiligte. Lust auf Neues mit Beteiligtsein verbinden Monitoring und Evaluation (institutionalisierte Reflexion/Überprüfung des Zielerreichungsgrades)

### Abschlussphase

In der Abschlussphase zeigte sich, wie wichtig die saubere Auflösung und Dokumentation sowie eine Reflektion des Reifegrades war. Zugleich wurde eine bewusste Haltung des Loslassens, des Empowerments und der Weitergabe etabliert, um den Weg hinein in den gelebten Alltag der Agilität zu erreichen.

**Tab. 9:** Cluster Dokumentation, Transfer sowie Auflösung

Dokumentation, Transfer sowie Auflösung	
Ziel(e)	Geordneter Abschluss des Vorhabens Lebendige Dokumentation als Ausgangslage für evolutionäre Organisationsentwicklung (OE) Auflösung des Agilitätsteam ermöglichen
Wirkungsebene	Bewusst
Umsetzung	Erstellung von Templates/Guidelines/Prozessdokumentation Wissensweitergabe durch verschiedene Kanäle Aufbau Kulturkreis, symbolische Stabsübergabe
Erkenntnisse	Verantwortung übernehmen Weitergeben und Befähigen mithilfe eines Rituals Transformationsgedanke ist Grundbestandteil der agilen Organisation

**Tab. 10:** Cluster Train the Trainer/Lernbegleitung

Train the Trainer/Lernbegleitung	
Ziel(e)	Verantwortung innerhalb der Kreise bei der Rolle „Lernbegleiter“ Selbstwirksamkeit und Vertrauen stärken – Empowerment
Wirkungsebene	Bewusst/unbewusst
Umsetzung	Mitarbeitende dürfen/sollen Lernbegleitungen/Unterstützung aktiv einholen Die Rollenverantwortungen der Lernbegleiter werden gestärkt Schrittweise Etablierung des Empowerments, über Team- zu Einzelentscheiden Sich selbst und andere reflektieren
Erkenntnisse	Verändertes „Führungsverständnis“ durch geteilte Führungsverantwortung Ex Führungskräfte bieten ihre Führungserfahrungen als Dienstleistung an

## Schlussfolgerungen

Dieser Artikel beschreibt die agile Transformation der Schweizer Stiftung IdeéSport und das Wagnis zu mehr Selbstorganisation. Die einst starre Organisation wurde aufgebrochen und anhand der vorgestellten Transformationsarchitektur durch das Kreismodell der kollegialen Führung ersetzt. Damit setzte IdeéSport die intrinsische Motivation ihrer Mitarbeitenden frei, sich schneller und gezielter um die Bedürfnisse ihrer Anspruchsgruppen zu kümmern. Die Mitarbeitenden arbeiteten auf der Basis von Vertrauen und in achtsamer Weise in einem Kreismodell enger zusammen und übernahmen mehr Verantwortung. Gemäss der Anspruchsgruppen und Mitarbeitenden war diese Transformation zu mehr Agilität erfolgreich, es wurden trotz intensiver Recherche keine Mängel direkt beobachtet. Heute verwendet IdéeSport nicht nur agile Prozesse, sondern nutzt ihre agile Denkweise als Innovationstreiber für interne Verbesserungen als auch für neue Kundenangebote.

Auch wenn sich die vorliegende Arbeit nur auf eine Non-Profit-Organisation (NPO) in der Schweiz fokussiert, verfolgte die Stiftung IdeéSport Absichten und Ziele, die ähnliche Organisationen mit ihr teilen. Um die Ergebnisse zu vergleichen und auf andere NPOs und verwandte Unternehmen zu übertragen, könnten in der Folge entsprechend quantitative Studien durchgeführt werden. Die Methodik dieser Arbeit bot einen tiefen Einblick in den Transformationsprozess einer NPO und ermöglichte die Formulierung von praktischen Vorschlägen in Form von empfohlenen Handlungsclustern. Dieser hätte mit mehr Zeit und vor Ort Präsenz noch intensiver begleitet werden können und gegebenenfalls dann ergänzende Details geliefert. Im Anschluss könnten weitere Forschungsarbeiten auch die organisations-übergreifenden Erfahrungen während und nach der Transformation untersuchen, einschliesslich der Auswirkungen auf die Anspruchsgruppen. Dies könnte zu neuen Erkenntnissen darüber führen, wie eine solche Transformation langfristig von den Mitarbeitenden und den betroffenen Interessengruppen wahrgenommen wird. Bei weiteren Begleitungen von Transformationsprozessen wären auch Fragestellungen zum Thema Auswahlentscheidungen für herangezogene Modelle spannend.

Zum Vorgehen bei der Durchführung einer agilen Transformation im Schweizer Stiftungswesen gibt es vielleicht keine allgemeingültige Empfehlung und nicht nur die eine, ideale und passende Lösung. Aber die Transformation selbst ist ein lebendiges Konstrukt; also empfiehlt es sich, diese agil zu gestalten. Das erfolgreiche, phasenweise, agile Vorgehen bei IdéeSport bestätigt dies auch in der Praxis. Die IdéeSport konnte eine höhere Innovationskraft mit neuen Angeboten realisieren, ohne dabei die Kontinuität im täglichen Betrieb zu beeinträchtigen. In der Initiierungsphase gelang es, die Dringlichkeit des Vorhabens zu betonen und die Aufmerksamkeit auf den Paradigmenwechsel zur Selbstorganisation zu lenken. Frühe Erfolgserlebnisse aus einem Pilotprojekt, die aktive Auseinandersetzung mit Spannungen und Widerständen sowie eine förderliche Lernkultur halfen bei der Umsetzung. Während der Transformationsphase war entscheidend, Betroffene zu Beteiligten zu machen und den agilen Mindset zu prägen. Die wichtigsten Erfolgsfaktoren waren eine achtsame Informations- und Kommunikationsstrategie, die persönliche Entwicklung durch zeitgerechte Qualifizierung und das gemeinsame Monitoring mit institutionalisierter Selbstreflexion. Am Ende diente die Abschlussphase dazu, das agile Vorgehen zu stabilisieren, Erfahrungen und Verantwortung zu teilen und den gemeinsamen Fortgang der Transformation durch kontinuierliches Lernen zu ermöglichen.

## References

[CR1] Anderson K, Uhlig J (2015) Das agile Unternehmen: wie Organisationen sich neu erfinden. Campus, Frankfurt am Main New York

[CR2] Antonello S (2020) Eine Stiftung geht neue Wege – und meistert Corona. Philanthr Aktuell 04/20:1–2

[CR3] Beck K, Beedle M, van Bennekum A (2001) Agile manifesto history. https://agilemanifesto.org/history.html. Zugegriffen: 15. Febr. 2021

[CR5] Eisenhardt KM, Graebner ME (2007) Theory building from cases: opportunities and challenges. Acad Manage J 50:25–32

[CR6] Findsrud R (2020) An agile approach to service innovation: creating valuable service innovation with agile resource integration. J Creat Value 6:190–207

[CR7] Futurum (2019) AGILITÄT IN KMU – Studie in der Schweiz

[CR8] Gloger B, Margetich J (2018) Das Scrum-Prinzip: agile Organisationen aufbauen und gestalten, 2. Aufl. Schäffer-Poeschel, Stuttgart

[CR9] Häusling A (2020) Agile Organisationen, 2. Aufl. Haufe Lexware

[CR10] He Z‑L, Wong P‑K (2004) Exploration vs. exploitation: an empirical test of the ambidexterity hypothesis. Organ Sci 15:481–494. 10.1287/orsc.1040.0078

[CR12] IdéeSport (2020) IdéeSport und Agilität: Eine Stiftung geht neue Wege

[CR13] Klimčíková V (2018) Führungsstile und Arbeitszufriedenheit

[CR14] Krstić M, Skorup A, Lapčević G (2018) Trends in agile innovation management. Int Rev. 10.5937/IntRev1804058K

[CR15] Langley A (1999) Strategies for theorizing from process data. Acad Manage Rev 24:691. 10.2307/259349

[CR16] Lehmann L, Engelhardt D, Wilke W (Hrsg) (2021) Kompetenzen für die digitale Transformation 2020: Digitalisierung der Arbeit – Kompetenzen – Nachhaltigkeit. Springer Vieweg, Berlin, Heidelberg

[CR17] Leikert-Boehm N, Sieber M, Kurz K, Russ C (2022) Learnings of an agile transformation in a non-profit organization case study. ZHAW, Winterthur

[CR18] Majkovic A‑L, Gundrum E, Benz SM et al (2019) IAP Studie 2019. Agile Arbeits- und Organisationsformen in der Schweiz. Ergebnisse der qualitativen Interviews. IAP Institut für Angewandte Psychologie der ZHAW Zürcher Hochschule für Angewandte Wissenschaften, Zürich

[CR19] Oestereich B, Schröder C (2017) Das kollegial geführte Unternehmen: Ideen und Praktiken für die agile Organisation von morgen. Vahlen

[CR20] O’Reilly CA, Tushman ML (2013) Organizational ambidexterity: past, present, and future. Acad Manag Perspect 27:324–338. 10.5465/amp.2013.0025

[CR21] Preußig J (2018) Agiles Projektmanagement: Agilität und Scrum im klassischen Projektumfeld, 1. Aufl. Haufe-Lexware, Freiburg

[CR22] Raisch S, Birkinshaw J, Probst G, Tushman ML (2009) Organizational ambidexterity: balancing exploitation and exploration for sustained performance. Organ Sci 20:685–695. 10.1287/orsc.1090.0428

[CR23] Renzl B, Mahringer C, Rost M, Scheible L (2021) Organizational agility: current challenges and future opportunities. J Competences Strateg Manage. 10.25437/jcsm-vol11-51

[CR24] Schein EH, Schein PA (2017) Organizational culture and leadership, 5. Aufl. Wiley, Hoboken

[CR25] Schwaber K, Sutherland J (2020) The Scrum guide

[CR26] Simsa R, Meyer M, Badelt C (2013) Führung in NPOs. In: Handbuch der Nonprofit-Organisation Strukturen und Management. Schäffer Poesche, S 359–377

[CR27] SwissFoundations (2021) Was ist eine Stiftung? – SwissFoundations. In: Stiftungsglossar. https://www.swissfoundations.ch/stiftungssektor/stiftungsglossar/. Zugegriffen: 9. Dez. 2021

[CR28] Tscherne P, Trapp D, Luge S (2018) Die agile Stiftung: Wie neue Organisationsformate für mehr Flexibilität und Klarheit sorgen. In: Berndt R, Kreutter P, Stolte S (Hrsg) Zukunftsorientiertes Stiftungsmanagement: Herausforderungen, Lösungsansätze und Erfolgsbeispiele. Springer, Wiesbaden, S 305–317

[CR4] Stiftung Schweiz Statistik (2021, November 17). [Stiftungen unter der Aufsicht der Eidg. Stiftungsaufsicht]. https://www.edi.admin.ch/edi/de/home/fachstellen/eidgenoessischestiftungsaufsicht/stiftungsverzeichnis/statistik.html

